# Supplementary material for: Robust perisomatic GABAergic self-innervation inhibits basket cells in the human and mouse supragranular neocortex
Source: eLife. 2020 Jan 9;9:e51691. doi: 10.7554/eLife.51691 (PMC6984819; doi:10.7554/eLife.51691)
Supplement: Supplementary file 1. — Columns from left to right show the figure in which the cell recording data are shown (cells showing no evidence for autapse are indicated as ‘no aut’), cell filing code, indication for successful recovery of cell with streptavidin visualization (strept) (NA indicates unsuccessful cell recovery), immunoreaction (positive as + and negative as -) for parvalbumin (pv) and vesicular GABA transported (vGAT), action potential inward current width (acw), firing frequency accommodation shown in cells where it was tested (first number shows firing frequency as ‘Hz’ during first 100 ms of a robust although not always maximal depolarizing pulse. Second number shows firing frequency accommodation ‘acc.’ = firing frequency at 400–500 ms during the depolarization divided by firing frequency during first 100 ms), and resting membrane potential (Em). Details of resected tissue are shown in blue, showing patient gender, age, hemisphere, cortical area and diagnosed primary pathology. There is ‘no info’ about exact neocortical area in some ventriculostomy operations. [file elife-51691-supp1.docx]

|  | **cell code** | **strept** | **immuno** | **acw**  **(ms)** | **firing freq. accomm.** | **Em (mV)** | **gender** | **age** | **hemisphere** | **neocortical area** | **diagnosed pathology** |
| --- | --- | --- | --- | --- | --- | --- | --- | --- | --- | --- | --- |
| **Fig 1 exp.** |  |  |  |  |  |  |  |  |  |  |  |
| GBZ | V041017_4 | strept | pv+, vGAT+ | 0.5 |  | -64 | female | 69 | right | occipital | subcortical neoplasia |
|  | V091117_1 | strept | pv+ | 0.5 | 160Hz/acc. 0.8 | -75 | female | 63 | right | temporal | hydrocephalus |
|  | V231117_2 | strept | pv+, vGAT+ | 0.7 |  | -73 | female | 68 | right | temporal | hydrocephalus |
|  | V071217_1 | strept | pv+ | 0.5 | 100Hz/acc. 1.1 | -70 | female | 76 | right | frontal | hydrocephalus |
|  | V130618_2 | NA |  | 0.5 |  | -63 | female | 35 | right | temporal | subcortical neoplasia |
|  |  |  |  |  |  |  |  |  |  |  |  |
| BAPTA | K190918_1 | NA |  | 0.4 | 170Hz/acc. 1.0 | -70 | male | 21 | right | temporo-occipital | subcortical cystic neoplasm |
|  | V190918_4 | strept | pv non concl. | 0.6 | 150Hz/acc. 0.8 | -70 | male | 21 | right | temporo-occipital | subcortical cystic neoplasm |
|  | V190918_5 | strept | pv non concl. | 0.5 |  | -73 | male | 21 | right | temporo-occipital | subcortical cystic neoplasm |
|  | V091018_1 | NA |  | 0.6 |  | -69 | female | 66 | left | frontal | cortical metaplasia |
|  | V171018_1 | NA |  | 0.6 | 150Hz/acc. 0.9 | -68 | male | 33 | right | frontal | hydrocephalus |
|  | V171018_2 | NA |  | 0.5 | 200Hz/acc. 0.9 | -76 | male | 33 | right | frontal | hydrocephalus |
|  |  |  |  |  |  |  |  |  |  |  |  |
| no aut | V041017_3 | strept | pv+, vGAT+ | 0.7 |  | -68 | female | 69 | right | occipital | subcortical neoplasia |
|  | V121217_1 | strept | pv+ | 0.65 |  | -74 | female | 68 | right | temporal | hydrocephalus |
| **Fig 2 and 3 exp.** |  |  |  |  |  |  |  |  |  |  |  |
|  | K151217_2 | strept | pv+ | 0.65 |  | -71 | female | 19 | right | frontal | subcortical neoplasia |
|  | K080917_1 | NA |  | 0.5 | 170Hz/acc. 0.76 | -63 | male | 45 | right | fronto-temporal | subcortical neoplasia |
|  | K121217_1 | strept | pv+, vGAT+ | 0.5 | 150Hz/acc. 0.8 | -81 | female | 68 | right | temporal | hydrocephalus |
|  | K150218_1 | strept | pv non concl. | 0.7 | 250Hz/acc. 0.6 | -76 | female | 67 | right | occipital | subcortical neoplasia |
|  | K151217_3 | strept | pv+, vGAT+ | 0.6 |  | -80 | female | 19 | right | frontal | subcortical neoplasia |
|  | K150917_2 | strept | pv+ | 0.6 |  | -68 | female | 63 | right | occipital | subcortical neoplasia |
|  | K270218_1 | strept | pv non concl. | 0.65 | 190Hz/acc. 1 | -78 | male | 34 | left | frontal | subcortical neoplasia |
|  | K260118_1 | strept | pv+ | 0.6 | 300Hz/acc. 0.83 | -83 | female | 54 | right | frontal | hydrocephalus |
|  | K071217_1 | strept | pv+ | 0.6 |  | -68 | female | 76 | right | frontal | hydrocephalus |
|  | K280218_1 | strept | pv+ | 0.5 |  | -84 | female | 41 | right | frontal | hydrocephalus |
|  | K121217_3 | strept | pv+ | 0.5 |  | -73 | female | 68 | right | temporal | hydrocephalus |
|  | K231117_3 | strept | pv non concl. | 0.65 | 130Hz/acc. 0.76 | -78 | female | 68 | right | temporal | hydrocephalus |
|  | k260118_2 | strept | pv+ | 0.65 | 260Hz/acc. 0.7 | -75 | female | 54 | right | frontal | hydrocephalus |
|  | k200218_6 | strept | pv+ | 0.45 | 310/acc. 0.8 | -62 | female | 54 | right | frontal | aneurysm |
|  |  |  |  |  |  |  |  |  |  |  |  |
| no aut | K280218_1 | strept | pv+ | 0.35 |  | -63 | female | 41 | right | frontal | hydrocephalus |
|  | K250118_1 | strept | pv+ | 0.35 |  | -75 | female | 50 | left | occipital | astrocytoma |
|  | K250118_2 | strept | pv+ | 0.6 | 220Hz/acc. 0.77 | -77 | female | 50 | left | occipital | astrocytoma |
|  | K200218_3 | strept | pv+ | 0.6 | 180Hz/acc. 0.8 | -74 | male | 56 | right | occipital | hydrocephalus |
|  | K200218_4 | strept | pv+ | 0.45 | 220Hz/acc. 0.86 | -61 | female | 54 | right | frontal | aneurysm |
|  | K270218_2 | strept | pv non concl. | 0.55 | 170Hz/acc. 0.76 | -71 | male | 34 | left | frontal | subcortical neoplasia |
|  | K231117_2 | strept | pv+ | 0.5 | 270Hz/acc. 0.6 | -88 | female | 68 | right | temporal | hydrocephalus |
|  | V220818_2 | strept | pv non concl. | 0.5 | 160Hz/acc. 0.8 | -71 | female | 26 | right | frontal |  |
|  | K250118_3 | strept | pv+ | 0.5 | 290Hz/acc. 0.9 | -71 | female | 50 | left | occipital | astrocytoma |
| **Suppl. fig. 2 exp.** |  |  |  |  |  |  |  |  |  |  |  |
|  | K231117_2 | strept | pv+ | 0.5 |  | -88 | female | 68 | right | temporal | hydrocephalus |
|  | K150218_1 | strept | pv non concl. | 0.65 |  | -70 | female | 67 | right | occipital | subcortical neoplasia |
|  | K151217_3 | strept | pv+, vGAT+ | 0.6 |  | -81 | female | 19 | right | frontal | subcortical neoplasia |
|  | K151217_2 | strept | pv+ | 0.65 |  | -74 | female | 19 | right | frontal | subcortical neoplasia |
|  | K121217_1 | strept | pv+, vGAT+ | 0.5 |  | -79 | female | 68 | right | temporal | hydrocephalus |
|  | K080917_1 | NA |  | 0.5 |  | -63 | male | 45 | right | fronto-temporal | subcortical neoplasia |
|  | K040915_2 | strept | pv+ | 0.5 | 240Hz/acc. 0.7 | -78 | male | 55 | right | frontal | subcortical neoplasia |
|  | K050615_1 | strept | pv non concl.,vGAT+ | 0.5 |  | -73 | male | 68 | right | temporal | glioblastoma grade IV. |
|  | K100417_1 | strept | pv+, vGAT+ | 0.4 | 210Hz/acc. 0.76 | -70 | female | 39 | right | frontal | glioblastoma grade IV. |
|  | K140415_2 | strept | pv and vGAT non concl. | 0.4 | 340Hz/acc. 0.8 | -89 | male | 18 | right | frontal | malignant germinoma, grade II. |
|  | K190315_1 | strept | pv non concl. | 0.4 | 360Hz/acc. 0.8 | -68 | male | 85 | left | fronto-temporal | hydrocephalus |
|  | K230615_1 | strept | pv+, vGAT+ | 0.4 | 270Hz/acc. 1 | -86 | female | 30 | no info | no info | hydrocephalus |
|  | K230915_1 | strept | pv+ | 0.5 | 330Hz/acc. 0.9 | -86 | female | 40 | right | frontal | anaplastic ependymoma |
|  | K230915_3 | strept | pv+, vGAT+ | 0.45 |  | -85 | female | 40 | right | frontal | anaplastic ependymoma |
|  | K250615_1 | strept | pv+ | 0.45 | 230Hz/acc. 1 | -93 | female | 10 | left | frontal | pilocytic astrocytoma |
|  | K250615_5 | strept | pv+ | 0.4 | 300Hz/acc. 0.9 | -85 | female | 10 | left | frontal | pilocytic astrocytoma |
|  | K260515_1 | strept | NA | 0.4 | 370Hz/acc. 0.8 | -83 | female | 31 | left | temporo-occipital | fibrillary astrocytoma |
|  | K280415_1 | strept | pv+, vGAT+ | 0.4 | 350Hz/acc. 0.8 | -84 | female | 28 | right | temporal | hydrocephalus |
|  | K280815_1 | strept | pv+ | 0.4 | 290Hz/acc. 0.8 | -80 | female | 58 | right | temporal | subarachnoid hemorrhage |
|  | K290415_2 | NA |  | 0.4 | 350Hz/acc. 1 | -91 | female | 32 | left | frontal | subcortical neoplasia |
| **pvBC-pvBC pairs** |  |  |  |  |  |  |  |  |  |  |  |
|  | K151217_3  presynaptic | strept | pv+, vGAT+ | 0.6 |  | -83 | female | 19 | right | frontal | subcortical neoplasia |
|  | postsynaptic | NA |  | 0.5 |  | -73 |  |  |  |  |  |
|  | K190619_1 mutual pair |  |  |  |  |  | female | 56 | left | temporal | subcortical neoplasia |
|  | cell 1 | NA |  | 0.55 |  | -64 |  |  |  |  |  |
|  | cell 2 | NA |  | 0.6 |  | -58 |  |  |  |  |  |
| **Fig 2 exp. In mouse** |  |  |  |  |  |  |  |  |  |  |  |
|  | K250618_1 | strept |  | 0.4 |  | -75 |  |  |  |  |  |
|  | K260618_2 | strept |  | 0.5 |  | -68 |  |  |  |  |  |
|  | K260618_5 | strept |  | 0.5 |  | -76 |  |  |  |  |  |
|  | K020718_3 | strept |  | 0.4 |  | -83 |  |  |  |  |  |
|  | K030718_1 | strept |  | 0.55 |  | -78 |  |  |  |  |  |
|  | K030718_3 | NA |  | 0.7 |  | -85 |  |  |  |  |  |
|  | K290618_2 | strept | pv+ | 0.6 |  | -75 |  |  |  |  |  |
|  | K290618_3 | strept | pv+ | 0.5 |  | -83 |  |  |  |  |  |
|  | K290618_4 | strept |  | 0.5 |  | -73 |  |  |  |  |  |
|  | V260618_1 | strept |  | 0.5 |  | -68 |  |  |  |  |  |
|  | V290618_4 | strept | pv+ | 0.7 |  | -86 |  |  |  |  |  |
| **Fig 4 exp.** |  |  |  |  |  |  |  |  |  |  |  |
|  | V101118_1 | strept | pv+ | 0.6 | 180Hz/acc. 0.76 | -76 | male | 20 | right | frontal | hydrocephalus |
|  | V150219_2 | strept | pv+ | 0.7 | 110Hz/acc. 0.8 | -64 | female | 67 | right | occipital | subcortical neoplasia |
|  | V150219_3 | strept | pv non concl. | 0.7 | 120Hz/acc. 0.8 | -64 | female | 67 | right | occipital | subcortical neoplasia |
|  | V260219_2 | strept | pv non concl. | 0.6 |  | -67 | male | 68 | no info | temporal | hydrocephalus |
|  | V050419_2 | strept | pv non concl. | 0.5 |  | -73 | female | 22 | right | frontal | hydrocephalus |
|  | V290319_1 | strept | pv non concl. | 0.5 | 210Hz/acc. 0.8 | -79 | male | 51 | no info | no info | hydrocephalus |
|  | V290319_2 | NA |  | 0.5 | 170Hz/acc. 0.9 | -68 | male | 51 | no info | no info | hydrocephalus |
|  |  |  |  |  |  |  |  |  |  |  |  |
| NonFSINs | V260219_1 | strept | pv-, vGAT+ | 1 | 80Hz/acc. 0.12 | -64 | male | 68 | no info | temporal | hydrocephalus |
|  | V120219_1 | strept | pv and vGAT non concl. | 0.8 |  | -68 | female | 66 | right | frontal | astrocytoma |
|  | V080119_2 | strept | vGAT+ | 0.9 |  | -81 | male | 49 | right | frontal | colloid cyst |
|  | v080119_1 | strept | pv-, vGAT+ | 0.8 | 130Hz/acc. 0.5 | -74 | male | 49 | right | frontal | colloid cyst |
|  | V160119_1 | strept | pv non concl. | 0.85 |  | -65 | male | 58 | left | frontal | epithelial carcinoma metaplasia |
|  | V131218_1 | strept | pv and vGAT non concl. | 0.9 |  | -71 | female | 79 | right | temporal | hydrocephalus |
|  | V050419_1 | strept | vGAT+ | 0.85 |  | -56 | female | 22 | right | frontal | hydrocephalus |
|  | V080518_1 | strept | pv and vGAT non concl. | 1 | 140Hz/acc. 0.7 | -81 | female | 68 | right | frontal | tumor |
| nonFSIN in results not in figures |  |  |  |  |  |  |  |  |  |  |  |
|  | V190918_3 | strept | pv- | 0.9 | 120Hz/acc. 0.5 | -73 | male | 21 | right | temporo-occipital | subcortical cystic neoplasm |
|  | V311017_2 | strept | pv- | 0.8 |  | -64 | female | 39 | right | frontal | glioblastoma |
|  | V091117_3 | strept | pv- | 1.2 |  | -63 | female | 63 | right | temporal | hydrocephalus |
|  | V100818_1 | strept | pv and vGAT non concl. | 0.9 |  | -59 | female | 46 | right | temporal | astrocytoma |
|  | V130618_1 | strept | pv- | 1.1 |  | -74 | female | 35 | right | temporal | subcortical neoplasia |
|  | V100518_2 | strept | pv and vGAT non concl. | 1 | 100Hz/acc. 0.6 | -76 | female | 73 | right | frontal | biopsy (MRI: multifocal lesions) |
|  | V220218_1 | strept | pv and vGAT non concl. | 0.85 |  | -74 | male | 42 | left | frontal | subcortical neoplasia |
|  | V091117_2 | strept | pv- | 0.9 | 90Hz/acc. 0.44 | -83 | female | 63 | right | temporal | hydrocephalus |
|  | K231117_4 | strept | pv- | 0.8 | 80Hz/acc. 0.5 | -88 | female | 68 | right | temporal | hydrocephalus |
|  | V151117_1 | NA |  | 0.9 | 60Hz/acc. 0.3 | -64 | female | 61 | right | frontal | fibrillic astrocytoma |
|  | K300119_1 | strept | pv and vGAT non concl. | 0.7 | 110Hz/acc. 0.5 | -83 | female | 69 | no info | no info | hydrocephalus |
|  | K260219_2 | strept | pv- | 0.7 |  | -65 | male | 68 | no info | temporal | hydrocephalus |
|  | V231117_1 | strept | pv and vGAT non concl. | 0.8 |  | -88 | female | 68 | right | temporal | hydrocephalus |
|  | K181017_1 | strept | pv and vGAT non concl. | 0.8 |  | -66 | female | 78 | right | frontal | hydrocephalus |
| **Fig 5 exp.** |  |  |  |  |  |  |  |  |  |  |  |
|  | V190619_2 | strept | pv+ | 0.6 | 230Hz/acc. 0.8 | -75 | female | 56 | no info | no info | hydrocephalus |
|  | V190619_3 | strept | pv+ | 0.55 | 170Hz/acc. 0.7 | -75 | female | 56 | no info | no info | hydrocephalus |
|  | V190619_4 | strept | pv+ | 0.6 | 150Hz/acc. 0.86 | -72 | female | 56 | no info | no info | hydrocephalus |
